# Supplementary material for: Dietary diversity and physical activity as risk factors of abdominal obesity among adults in Dilla town, Ethiopia
Source: PLoS One. 2020 Jul 30;15(7):e0236671. doi: 10.1371/journal.pone.0236671 (PMC7392300; doi:10.1371/journal.pone.0236671)
Supplement: S1 Appendix — (PDF) [file pone.0236671.s001.pdf]

S1 Appendix: Wealth index assets and utilities, Dilla town, Ethiopia, 2018

Wealth index assets and utilities			
Does the household have any of the following assets and utilities? (Circle)		Yes	No
1	Television	1	0
2	Refrigerator	1	0
3	Stove (Cylinder/Electric)	1	0
4	CD player	1	0
5	Table	1	0
6	Bicycle	1	0
7	Motor cycle	1	0
8	Animal-drawn cart	1	0
9	Bajaj	1	0
10	Bed with Cotton/Sponge/Spring mattress	1	0
11	Car	1	0
12	Computer	1	0
13	Sofa (chair)	1	0
14	House	1	0
15	Mobile Phone	1	0
16	Video camera	1	0
17	Radio	1	0
18	Washing machine	1	0
